# Preconceptional and prenatal exposure to diurnal temperature variation increases the risk of childhood pneumonia

**DOI:** 10.1186/s12887-021-02643-x

**Published:** 2021-04-21

**Authors:** Xiangrong Zheng, Jian Kuang, Chan Lu, Qihong Deng, Haiyu Wu, Rachael Gakii Murithi, McSherry Brownel Johnson, Wang Peng, Maolan Wu

**Affiliations:** 1 Department of Pediatrics, XiangYa Hospital, Central South University, Changsha, Hunan China; 2 XiangYa School of Public Health, Central South University, 410078 Changsha, Hunan China; 3 School of Energy Science and Engineering, Central South University, Changsha, Hunan China; 4 School of Public Health, Zhengzhou University, Zhengzhou, Henan China; 5 XiangYa School of Medicine, Central South University, Changsha, Hunan China

**Keywords:** Diurnal temperature variation, Preconceptional, Pregnancy, Trimester, Childhood pneumonia

## Abstract

**Background:**

Pneumonia is the leading cause of death and hospitalization among young children worldwide, but its risk factors remain unclear.

**Objective:**

To evaluate the effect of maternal exposure to diurnal temperature variation (DTV) during preconceptional and prenatal periods on childhood pneumonia.

**Methods:**

A retrospective cohort study by case-control design was conducted for pneumonia (*N* = 699) and normal (*N* = 811) children under age of 14 who were enrolled in XiangYa Hospital, Changsha, China from May 2017 to April 2019. Demographic data including gender, age, birth season, gestational age, parity, mode of delivery, and parental atopy were collected from the electronic medical records in the hospital system. We obtained the data of daily DTV in Changsha during 2003–2019 from China Meteorological Administration. Maternal exposure to DTV during preconceptional and prenatal periods was respectively calculated by the average of daily DTV during one year and three months before conception and entire pregnancy as well as the three trimesters. The association between maternal exposure to outdoor DTV and childhood pneumonia was analyzed by multiple logic regression model.

**Results:**

We found that childhood pneumonia was significantly associated with exposure to an increase in DTV during one year before conception and entire pregnancy, with ORs (95 % CI) = 2.53 (1.56–4.10) and 1.85 (1.24–2.76). We further identified a significant risk of pneumonia of DTV exposure during the first and second trimester of pregnancy. Sensitivity analysis showed that boys were more susceptible to the effect of prenatal exposure to outdoor DTV during pregnancy particularly in the first two trimesters compared to girls.

**Conclusions:**

Preconceptional and prenatal exposure to DTV plays an important role in development of childhood pneumonia, especially during the first and second trimesters of pregnancy.

## Background

Pneumonia is the leading cause of death and hospitalization among children under age of 5 worldwide [1, 2]. Although pneumonia treatment has been improved significantly during past decades, the incidence of pneumonia in childhood remains high, especially in developing countries [3]. According to a recent epidemiological report, pneumonia has resulted in 808,694 deaths of children in 2017, which accounts for 15 % of all deaths in children under 5 years [4]. Notably, China has witnessed a very high prevalence of childhood pneumonia over recent years [5]. Pneumonia not only influences the growth and development of children’s lung, but also causes a heavy burden on family economy and public health [6]. Therefore, it is crucial to investigate the risk factors contributing to the frequent occurrence of childhood pneumonia, which would have a great implication for its effective reduction and early prevention.

Recently, there is increasing awareness that climatic variation significantly impacts childhood respiratory health. Pneumonia, as one of the most adverse infectious disease among children, has been suggested to be closely related to a continuous climate change [7, 8]. Mounting evidences indicate that children suffer most from climate change due to their susceptibility for the extreme temperature and the dramatic weather change [9, 10]. Some recent epidemiological studies from different regions have found an association between outdoor temperature and childhood pneumonia [11, 12]. Diurnal temperature variation (DTV), reflecting a degree in the daily temperature changes, has been found to be correlated with both mortality and morbidity of respiratory diseases [13–18]. Particularly, one of our recent cohort studies suggests that outdoor DTV is an important risk factor for pneumonia development in young children [19].

Early life exposure to harmful environmental factors could not only have an adverse effect on organ development and childhood growth, but also plays a critical role in the onset and progression of diseases in adulthood and has a long-term impact in the later life [20, 21]. Some studies have suggested that early-life exposure to temperature and DTV contributed to an increased risk of childhood respiratory diseases [19, 22–24]. Although a few studies have linked maternal exposure to traffic-related air pollution (TRAP) during preconceptional period with increased risk of allergic diseases in preschool children [25, 26]. Thus, maternal exposure to environmental factors before pregnancy could play an important role in the development of health outcomes during postnatal period. However, the effect of preconceptional exposure to outdoor DTV on respiratory outcomes, such as childhood pneumonia, have scarcely been investigated.

Due to the lack of studies on early life exposure to climatic change particularly before birth and children’s health, we hypothesized that exposure to outdoor DTV during preconceptional and prenatal periods may be associated with later development of childhood pneumonia. To test this hypothesis and identify the critical timing window(s), we performed a large retrospective cohort study via case-control design in Changsha, China.

## Methods

### Study population

A retrospective cohort study by case-control design was conducted in the Department of Pediatrics, XiangYa hospital, Central South University in Changsha, China from May 2017 to April 2019. We recruited 699 children under 14 years old who were diagnosed with pneumonia and set them as the case group, and 811 healthy children who registered for physical examination as the control group. Children were excluded if they had abnormal congenital development in the airway and lung parenchyma, chronic lung disease, obstruction in the airway or compression outside the tube, congenital heart disease, tuberculosis, heart, liver and kidney diseases, or a known diagnosis of immunodeficiency.

The case group consisted of children with a diagnosis of pneumonia made in accordance with the following WHO diagnosis criteria [27]. Cough or difficult breathing plus at least one of the following signs: (1) fast breathing (rate > 60 breaths per minute if aged < 2 months, > 50 breaths per minute if aged 2–11 months, and > 40 breaths per minute if aged 12–59 months); or (2) lower chest wall indrawing. In addition, either crackles or pleural rub may be present on chest auscultation. The control group included healthy children who didn’t have a history of pneumonia diagnosis based on WHO criteria at the time of recruitment.

### Demographic data

We collected the demographic data from the electronic medical records system in the hospital, including children’s gender, age, birth season, gestational weeks, parity, mode of delivery, and parental atopy. Parental atopy was defined by the presence of a history of any allergic diseases among child’s mother and/or father.

### Exposure assessment

#### Exposure timing windows

Exposure timing-windows included preconceptional and prenatal periods in this study. The preconceptional exposure involved in two timing windows: one year before conception and three months before conception. The prenatal period was defined from the first day of the mother’s last menstrual period to the delivery day. The prenatal exposure was further divided into three trimesters: the first trimester (from the 1st to 12th weeks of gestation), second trimester (the 13th to 27th weeks of gestation), and third trimesters (from the 28th gestational week to the birthday of the child).

#### Personal exposure to diurnal temperature variation (DTV)

We obtained the data of daily temperature (including the mean, maximum, and minimum temperatures) at ten different monitoring stations in Changsha during 2003–2019 from China Meteorological Administration: Kaifu district, Yuhua district, Furong district, Liuyang county, Mapoling, Ningxiang county, Tianxin district, Wangcheng district, Yuelu district, and Changsha county. The diurnal temperature variation (DTV) was calculated as the difference between the daily maximum and minimum temperature. Children’s exposure was estimated by DTV at the station where their residence was located. Then, the individual exposure to outdoor DTV for each child was calculated as follows: (1) Preconceptional exposure to DTV was calculated as the average of daily DTV during one year and three months before conception, (2) Prenatal exposure to DTV was calculated by the average of daily DTV during the period from the mother’s last menstrual period to the delivery day; (3) The exposure to DTV in the first, send, and third trimester was respectively calculated as the average of daily DTV during the periods of 1st − 12th gestational weeks, 13th − 27th gestational weeks, and from the 28th gestational week till the date of birth.

#### Exposure to outdoor air pollution

Exposure to outdoor air pollution was considered as an important confounding variable for pneumonia risk in this study. We obtained daily 24 h-averaged concentrations of three main air pollutants, including nitrogen dioxide (NO_2_), sulfur dioxide (SO_2_), and particulate matter ≤ 10 μm in diameter (PM_10_), from 7 municipal air quality monitoring stations in Changsha from 2003 to 2019. Individual exposure to ambient air pollution during different timing windows was estimated by the inverse distance weighted (IDW) method described in our previous work [28].

### Statistical analysis

Statistical analyses were performed by SPSS software (version 23.0, SPSS Inc., Chicago, USA). The relationship between exposure to outdoor DTV during preconceptional and prenatal periods and childhood pneumonia was assessed by using multiple logistic regression model, with adjusting potential variables in Table 1 and the three air pollutants (NO_2_, SO_2_, and PM_10_). The associations in regression analysis was calculated by odds ratio (OR) of 95 % confidence interval (95 % CI). In our study, OR (95 % CI) was estimated by per 1 °C increase in exposure to outdoor DTV. The data were drawn into a figure by Origin software (version: OriginPro 2018 C). *P*-value ≤ 0.05 was considered as statistically significant.

**Table 1:** Demographic information of covariates among children with (case) and without (control) doctor-diagnosed pneumonia (*n* = 1,510)

	Total		Case		Control		
	N	(%)	N	(%)	N	(%)	*P*-value
Total	1,510	(100.0)	699	(46.3)	811	(53.7)	—
**Sex**							**< 0.001**
Boys	865	(57.3)	436	(50.4)	429	(49.6)	
Girls	645	(42.7)	263	(40.8)	382	(59.2)	
**Age (years)**							**< 0.001**
< 1	402	(26.6)	334	(83.1)	68	(16.9)	
= 1	323	(21.4)	130	(40.2)	193	(59.8)	
> 1	785	(52.0)	235	(29.9)	550	(70.1)	
**Birth season**							**0.048**
Spring	342	(22.6)	138	(40.4)	204	(59.6)	
Summer	392	(26.0)	184	(46.9)	208	(53.1)	
Autumn	422	(27.9)	197	(46.7)	225	(53.3)	
Winter	354	(23.4)	180	(50.8)	174	(49.2)	
**Parity**							**0.046**
1st	1,025	(67.9)	454	(44.3)	571	(55.7)	
2nd – 5th	478	(31.7)	238	(49.8)	240	(50.2)	
**Gestational age (weeks)**							**< 0.001**
< 37	92	(6.1)	62	(67.4)	30	(32.6)	
≥ 37	1,418	(93.9)	637	(44.9)	781	(55.1)	
**Mode of delivery**							**< 0.001**
Natural labour	863	(57.2)	360	(41.7)	503	(58.3)	
Caesarean birth	641	(42.5)	333	(52.0)	308	(48.0)	
**Parental atopy**							**< 0.001**
No	1,432	(94.8)	646	(45.1)	786	(54.9)	
Yes	78	(5.2)	53	(67.9)	25	(32.1)	

Sum of the number is not 1,510 due to missing data. The *p*-values < 0.05 were in bold

## Results

Data on the demographics and prevalence of pneumonia stratified by the covariates are given in Table 1. The total material (*N* = 1,510) included 699 children with pneumonia and 811 healthy children without pneumonia or other respiratory diseases. We conducted one-way analysis of variance to examine the association between childhood pneumonia and the considered potential covariates. We found that boys, younger children (< 1 year old), children with gestational age < 37 weeks (preterm birth), delivery mode of caesarean, and parental atopy had a significantly higher prevalence of pneumonia than girls, older children, children with gestational age ≥ 37 weeks, natural delivery mode and without parental atopy, and the p-values were statistically significant (*p* < 0.05). Furthermore, we observed that the prevalence of pneumonia was significantly higher in children born in winter compared to the other seasons.

Data on ambient DTV during different periods between the case and control group were given in Table 2. The average of individual exposure to DTV during one year before conception and three months before conception were both 7.3°C in the case group and 7.2 and 7.1°C in the control group, with a statistically significant difference (*p* values < 0.001). The mean DTV exposure during entire pregnancy was significantly larger in children with pneumonia (7.3°C) than that in healthy children (7.0°C), *p* value < 0.001. Moreover, we observed that exposure to DTV during all the three trimesters of pregnancy were significantly larger in children with pneumonia than healthy children (*p* < 0.001)

**Table 2:** Descriptive statistics for outdoor temperature and DTV during different time windows attributed to the children (*n* = 1,510)

	Total		Case		Control		*P*-value
	Mean	(SD)	Mean	(SD)	Mean	(SD)	
**Preconceptional**							
1 year before conception	7.2	(0.5)	7.3	(0.4)	7.2	(0.5)	**< 0.001**
3 months before conception	7.2	(0.8)	7.3	(0.7)	7.1	(0.8)	**< 0.001**
**Prenatal**							
1st trimester	7.2	(0.8)	7.3	(0.7)	7.0	(0.8)	**< 0.001**
2nd trimester	7.1	(0.7)	7.3	(0.7)	6.9	(0.7)	**< 0.001**
3rd trimester	7.1	(0.7)	7.3	(0.7)	6.9	(0.7)	**< 0.001**
Entire pregnancy	7.1	(0.5)	7.3	(0.5)	7.0	(0.5)	**< 0.001**

DTV (°C) = Tmax - Tmin. The *p*-values < 0.05 were in bold

We further evaluated the relationship between DTV exposure during preconceptional and prenatal periods and childhood pneumonia by using multiple logistic regression model, with an adjustment of the potential variables (Table 3). We found that maternal exposure to DTV was significantly associated with childhood pneumonia, particularly for a long-term exposure, with the adjusted OR (95 % CI) of 2.53 (1.56–4.10) for per 1°C increase in DTV exposure during one year before conception. We also detected significant associations of childhood pneumonia with prenatal exposure to outdoor DTV during entire pregnancy especially in the first and second trimester, with adjusted ORs (95 % CI) = 1.85 (1.24–2.76), 1.63 (1.32-2.00) and 1.43 (1.12–1.81) respectively.

**Table 3:** Odds ratio (95 %CI) of childhood pneumonia for exposure to outdoor DTV during different timing-windows(*n* = 1,510)

	Crude OR	Adjusted model I^a^	Adjusted model II^b^
**Preconceptional**			
3 months before conception	1.40 (1.22, 1.61)***	1.98 (1.67, 2.34)***	1.15 (0.91, 1.46)
1 year before conception	1.86 (1.48, 2.32)***	10.21 (7.08, 14.71)***	2.53 (1.56, 4.10)***
**Prenatal**			
1st trimester	1.72 (1.50, 1.97)***	2.30 (1.94,2.72)***	1.63 (1.32, 2.00)***
2nd trimester	1.99 (1.71, 2.33)***	2.55 (2.12,3.07)***	1.43 (1.12, 1.81)**
3rd trimester	1.88 (1.62, 2.18)***	2.29 (1.91,2.73)***	1.20 (0.96, 1.51)
Entire pregnancy	4.45 (3.52, 5.6)***	8.36 (6.21,11.27)***	1.85 (1.24, 2.76)**

OR (95% CI) was estimated by 1°C increase in outdoor DTV during each time window.

^a^Model I was adjusted for the covariates in Table 1.

^b^Model II was further adjusted for outdoor air pollution exposure during each time window based on model I.

* *p*≤0.05.

** *p*≤0.01.

*** *p*≤0.001

**Fig. 1:**
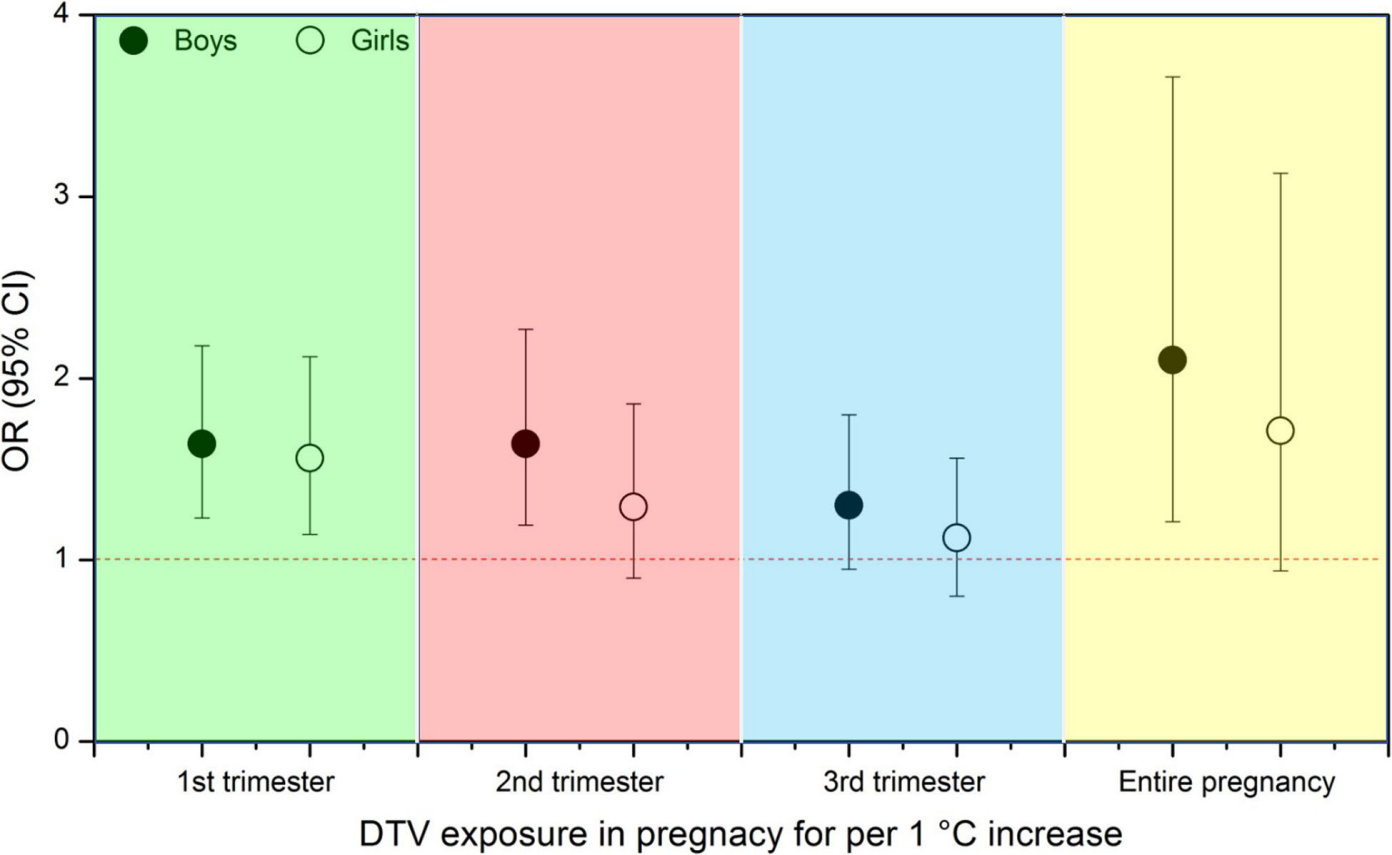
Odds ratio (95 %CI) of childhood pneumonia for exposure to diurnal temperature variation (DTV) stratified by gender during pregnancy (*n* = 1,510). ORs was adjusted for all covariates in Table 1 and outdoor air pollutants (PM_10_, SO_2_ and NO_2_)

Figure 1 showed pneumonia risk of prenatal DTV exposure stratified by children’s gender. We observed that the odds of pneumonia due to DTV exposure during the second trimester and entire pregnancy were significant only in boys but not in girls. Furthermore, the association between DTV exposure in the first trimester was greater among boys than that among girls.

## Discussion

In this retrospective cohort study, we are the first to find a significant association between maternal exposure to outdoor DTV during one year before conception and childhood pneumonia. We also observed that prenatal exposure to DTV during pregnancy, especially in the first and second trimesters, was significantly associated with an increased risk of childhood pneumonia, supporting the hypothesis of “fetal origin of diseases”. Our sensitivity analysis indicated that boys were more vulnerable to the pneumonia risk of prenatal exposure to DTV than girls.

Previous studies have suggested that DTV exposure may play a significant role in the increased risk of respiratory diseases [29–31], including asthma, allergy and respiratory tract infection (RTI) among young children [32–34]. However, the evidence in the association between DTV and childhood pneumonia is still controversial [19, 35]. Our study newly indicates that early-life exposure to a large DTV could be a risk factor contributing to the development of childhood pneumonia.

As far as we know, the present study is the first to find that increased maternal DTV exposure during the year prior conception has an adverse effect on the later development of pneumonia in children. Previous studies mainly associated preconceptional exposure to air pollution with childhood health problems. A cohort study in China suggested that preconceptional exposure to traffic- and industry-related air pollutants was significantly related withchildhood asthma [26]. NO_2_ exposure during three months before conception was reported to be linked with eczema risk in children [25]. Another recent retrospective study in US proved a significant relationship between preconceptional exposure to NO_X_ and SO_2_ and an increased risk of gestational diabetes mellitus (GDM) [36]. Furthermore, a multi-site study found that preconceptional exposure to PM_10_, SO_2_ and CO was linked with increased risk of oral cleft [37]. Therefore, it is reasonable to believe that preconceptional exposure to DTV significantly increased the risk of childhood pneumonia, which may indicate a hypothesis of “pre-fetal origin of childhood infection”.

Professor David Barker, a British epidemiologist, first proposed the theory of the development origins of health and diseases (DOHaD): early life (prenatal and early postnatal periods) exposure to environmental factors affects the plasticity of development, leading to the development and/or deterioration of various complex diseases in adulthood. Therefore, early life was considered as a critical timing window of environmental exposure which is related to human health. In this study, we observed that prenatal exposure to a large DTV was a significant risk factor for pneumonia development in children, which is in line with a recent cohort study from China [19]. We further identified that the first and second trimester of pregnancy was the critical time windows for DTV exposure contributing to the development of childhood pneumonia, which was scarcely observed in previous literatures. Exposure to a large DTV in the first trimester of pregnancy was found to be associated with frequent common colds among preschool children in China [22]. One of our recent studies have indicated a significant association between exposure to industrial air pollutant SO_2_ during the first trimester of pregnancy and increased risk of childhood ear infection [38]. The mechanisms underlying the association between childhood pneumonia and prenatal exposure to DTV during the first and second trimesters of pregnancy remain unclear. It was suggested that lung development starts at about the 4th gestational week, nad the airway branching morphogenesis occurs in the first two trimesters of gestation [39]. Hence, long-term exposure to environmental factors in utero may lead to an improper development of lung at various degree, which could affect postnatal development of lung function and induce a risk of respiratory diseases later in life. However, the pathogenic mechanisms underlying childhood pneumonia risk of intrauterine exposure to DTV warrants further investigations.

A recent study [13] and one of our recent work [19] indicated that boys were more susceptible to the effect of prenatal exposure to DTV in pregnancy on pneumonia risk compared to girls. According to the reports from Global Burden of Disease Study 2017 (GBD 2017), the prevalence of respiratory tract infections in males was significantly higher than females [40]. Some evidence suggested that the gender difference of environmental susceptibility was mainly related to immune response [41]. In general, females are more responsive to pathogenic stimuli and vaccines than males, and thus females are more resistant to infectious diseases [42]. Whereas, the mechanisms in the sex-related susceptibility in the effect of DTV exposure on childhood infections including pneumonia remain unclear, which is profound to further study.

Our study had several limitations for the data collection and analysis. Firstly, the data of personal exposure to outdoor air temperature was only obtained from one meteorological station, this may result in exposure misclassification. In spite of the fact that environmental temperature changes in different areas of one city (Changsha) are small, this may lead to exposure classification errors, and using temperature exposure with high spatial resolution to capture the temperature difference in the areas of inner-city may perform a better exposure assessment [43]. Secondly, indoor air temperature was not considered in the present study. However, as people especially pregnant women spend most of their time indoors, the long-term exposure to indoor air temperature may impact the health condition among pregnant mothers and young childhood. Thirdly, other meteorological parameters including relative humidity, wind speed, and precipitation were not included in our study.These climatic factors may affect air temperature or temperature change, and thus their potential influence should not be ignored. At last, we didn’t consider the possibility of the change of residence of the mother during the preconception or prenatal period, however, most of them would like to stay at home and always like to work near their residence because it may waste a lot time if they live far from their working place due to rush hour in Chinese cities.

## Conclusions

This retrospective cohort study via case-control design reveals that preconceptional (one year before conception) and prenatal exposure to DTV was significantly associated with childhood pneumonia. We identified the first two trimesters of pregnancy as the critical time windows. Our results have a clinical significance in assessing the onset and development of pneumonia. Our study also provide a novel strategy for effective reduction and early prevention of childhood pneumonia, i.e., reducing outdoor activities among women preparing for pregnancy when the temperature changes sharply, and avoiding extra exposure to a large variation in daily temperature during pregnancy especially in the first and second trimesters.

## Data Availability

The data sets used and/or analyzed during the current present study are available from the corresponding author on reasonable request.

## Abbreviations

DTV: Diurnal temperature variation
IDW: Inverse distance weighted
